# Isolation and Characterization of Extracellular Vesicles in Human Bowel Lavage Fluid

**DOI:** 10.3390/ijms24087391

**Published:** 2023-04-17

**Authors:** Marina Alorda-Clara, Jose Reyes, Marita Grimanesa Trelles-Guzman, Monica Florido, Pilar Roca, Daniel Gabriel Pons, Jordi Oliver

**Affiliations:** 1 Grupo Multidisciplinar de Oncología Traslacional, Institut Universitari d’Investigació en Ciències de la Salut (IUNICS), Universitat de les Illes Balears, E-07122 Palma, Spain; marina.alorda@uib.es (M.A.-C.); jose.reyes@hcin.es (J.R.); marita.trelles@hcin.es (M.G.T.-G.); pilar.roca@uib.es (P.R.); jordi.oliver@uib.es (J.O.); 2 Instituto de Investigación Sanitaria Illes Balears (IdISBa), Hospital Universitario Son Espases, Edificio S, E-07120 Palma, Spain; 3 Servicio Aparato Digestivo, Hospital Comarcal de Inca, E-07300 Inca, Spain; monica.florido@hcin.es; 4 Ciber Fisiopatología Obesidad y Nutrición (CB06/03), Instituto Salud Carlos III, E-28029 Madrid, Spain

**Keywords:** bowel lavage fluid, extracellular vesicles, colorectal cancer, ultracentrifugation

## Abstract

Colorectal cancer (CRC) is the third most common cancer worldwide and is detected in late stages because of a lack of early and specific biomarkers. Tumors can release extracellular vesicles (EVs), which participate in different functions, such as carrying nucleic acids to target cells; promoting angiogenesis, invasion, and metastasis; and preparing an adequate tumor microenvironment. Finally, bowel lavage fluid (BLF) is a rarely used sample that is obtained during colonoscopy. It presents low variability and protein degradation, is easy to handle, and is representative of EVs from tumor cells due to proximity of the sample collection. This sample has potential as a research tool and possible biomarker source for CRC prognosis and monitoring. In this study, EVs were isolated from human BLF by ultracentrifugation, then characterized by transmission electron microscopy and atomic force microscopy. EV concentration was determined by nanoparticle tracking analysis, and tetraspanins were determined by Western blot, confirming correct EV isolation. RNA, DNA, and proteins were isolated from these EVs; RNA was used in real-time PCR, and proteins were used in an immunoblotting analysis, indicating that EV cargo is optimal for use and study. These results indicate that EVs from BLF can be a useful tool for CRC study and could be a source of biomarkers for the diagnosis and monitoring of CRC.

## 1. Introduction

Colorectal cancer (CRC) is the third most common cancer worldwide and the second leading cause of cancer death [1]. It is known that an early diagnosis of CRC improves patient survival, decreasing mortality; however, at present, CRC is usually detected in late stages because of the lack of early biomarkers and detection techniques [2]. Even so, there are different diagnostic techniques that are used in the clinical setting, the most commonly used of which are diagnostic imaging techniques, such as colonoscopies, positron emission tomography, computed tomography, and nuclear magnetic resonance; however, all these techniques are invasive for the patients [3,4]. There are some less invasive techniques; however, these are non-specific for CRC because of their presence in other pathologies, in addition to their presence in late stages of CRC, such as carcinoembryonic antigen (CEA) detection in plasma, as well as the detection of carbohydrate antigen 19-9 (CA19-9), tumor-associated glycoprotein 72 (TAG-72), and tissue polypeptide-specific antigen (TPS) in serum [4]. Finally, stool is also used for CRC diagnosis; the most used diagnosis techniques are fecal occult blood testing, which presents high sensitivity and specificity but also a high number of false positives [5,6]; the detection of fecal calprotectin, which presents low sensitivity [7]; and the detection of changes in DNA, which is stable in stool [4].

Minimal information of studies of extracellular vesicles (MISEV) describes the extracellular vesicles (EVs) as “particles naturally released from the cell that are delimited by a lipid bilayer and cannot replicate” [8]. EVs can participate in several diseases, such as cancer, diabetes mellitus, cardiovascular diseases, neurodegenerative diseases, and autoimmune diseases [9]. It is known that CRC cells release EVs starting in the early stages; for this reason, EVs can be used as a possible source of prognostic and monitoring biomarkers for this cancer type [2]. EVs present some advantages over other diagnostic techniques; for example, they are easy to obtain in high concentrations, have a long half-life, can be collected non-invasively, are stabilized and protected by the lipid bilayer, and their cargo can reflect tumor status [3,10,11] because it is based on the genome, transcriptome, and secretome of origin tumoral cells [10,12]. EVs can participate in different functions: debris removal, carrying DNA, RNA and protein targeting of cells, tumor microenvironment formation, tumor formation and progression, angiogenesis, invasion, metastasis, chemoresistance, drug resistance, recrudescence, tissue repair, tumoral therapy, and vaccination [2,9,10,12,13,14,15].

Bowel lavage fluid (BLF) is obtained during colonoscopies by direct application of saline serum to the injury area in the colon, enriching it with injury-area cells [16]. Few studies have been conducted using this sample type for CRC research, with most studies focusing on the presence of genomic biomarkers such as an increase in p53 and K-ras mutations, an increase in mutations of the TGFβ type II receptor and APC [17,18], the presence of aberrant methylation in CpG islands in a gene panel (miR-124-3p, LOC86758, and SFRP1) [19], and the presence of syndecan-2 methylation [20]. BLF presents some advantages over other samples types such as stool because it is subject to less bacterial and food interference, is easier to handle, presents less variability because of water quantity and time in the bowel, and is less subject to protein degradation [16]. Nowadays, BLF is rarely used, but this type of sample has a great potential as a CRC research tool and could be studied as possible biomarker source for CRC [16].

The aim of this study was to evaluate BLF as a research tool for the study of CRC through EV isolation in this sample type.

## 2. Results

### 2.1. Extracellular Vesicles Can Be Isolated from Bowel Lavage Fluid

**Figure 1:**
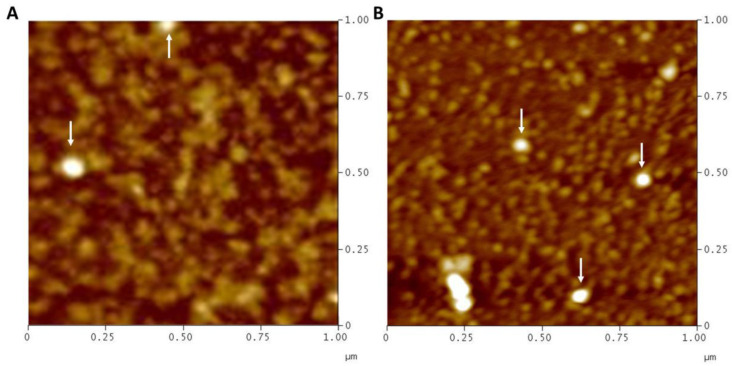
Extracellular vesicle visualization in healthy (**A**) and cancer (**B**) samples by atomic force microscopy. White arrows point to extracellular vesicles.

**Figure 2:**
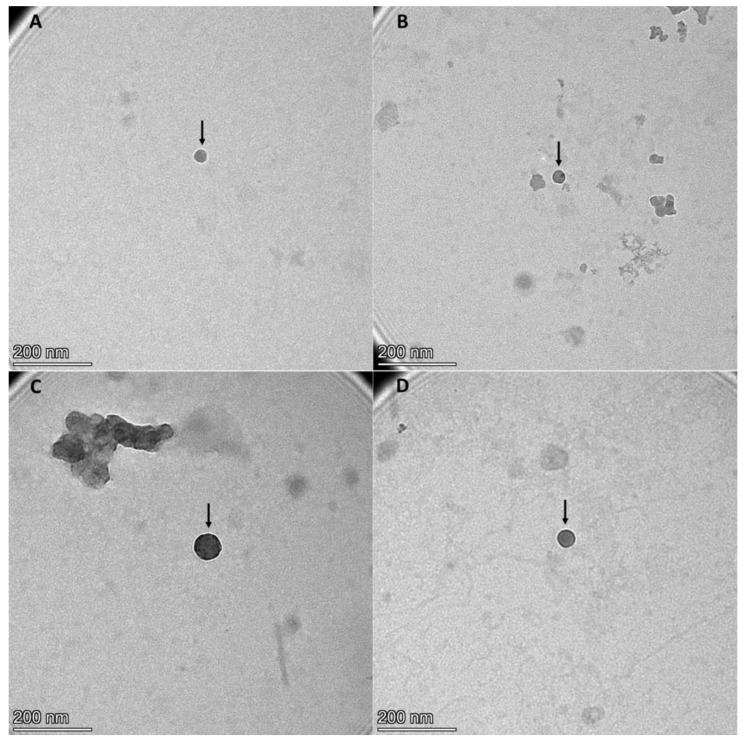
Extracellular vesicle structural and morphological visualization in healthy (**A**), low-risk (**B**), high-risk (**C**), and cancer (**D**) samples by transmission electron microscopy. Black arrows point to extracellular vesicles. *n* = 16, divided into 4 pools of 4 samples each; 1 pool per sample type.

**Figure 3:**
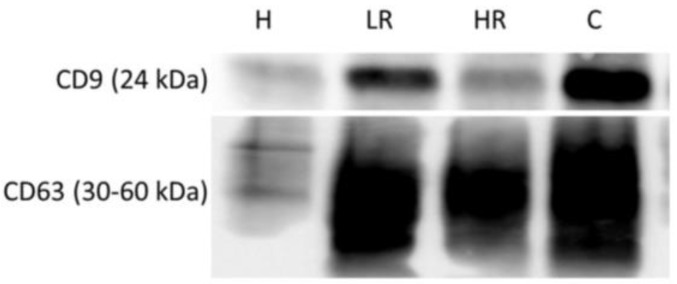
Extracellular vesicle characterization in healthy, low-risk, high-risk, and cancer samples. Tetraspanins (CD9 and CD63) representative bands and their molecular weights determined by Western blot. **H:** healthy samples; **LR:** low-risk samples; **HR:** high-risk samples; **C:** cancer samples.

**Figure 4:**
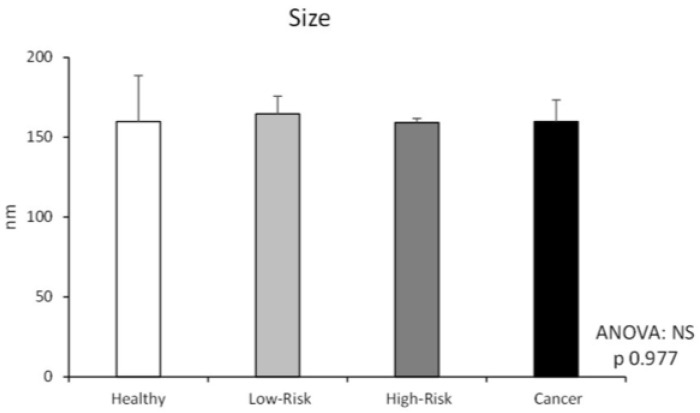
Extracellular vesicle characterization in healthy, low-risk, high-risk, and cancer samples. Extracellular vesicle size (nm) was determined by nanoparticle tracking analysis, and values are represented as mean ± SD. **NS**: non-significant differences (*p* 0.977), (ANOVA; *p* < 0.05). *n* = 48; for each sample type, *n* = 12 divided into 3 pools of 4 samples each.

After EVs isolation from BLF samples by ultracentrifugation method, EVs were characterized by observation under AFM and TEM microscopes (Figure 1 and Figure 2). Furthermore, two different tetraspanins (CD9 and CD63) determined by Western blot were present in EVs from BLF (Figure 3). Finally, the size determination and EVs concentration determined by NTA (Figure 4 and Table 1) indicated no significant differences between groups.

### 2.2. RNA Can Be Isolated from Extracellular Vesicles from Bowel Lavage Fluid and Can Be Used for Real-Time PCR

RNA can be isolated from EVs from BLF using TRI reagent LS (Table 2). Healthy samples presented an RNA concentration of 17.2–3311 ng/mL, low-risk samples presented a concentration of 46.7–460 ng/mL, high-risk samples presented a concentration of 9.5–2343 ng/mL, and cancer samples presented a concentration of 39.5–8242 ng/mL. The results show non-significant differences between groups.

This RNA can be used for gene expression determination by real-time PCR. The study of B2M expression levels (Table 3) presented non-significant differences between groups.

### 2.3. DNA Can Be Isolated from Extracellular Vesicles from Bowel Lavage Fluid

DNA can be isolated from EVs from BLF using TRI reagent LS (Table 2). Healthy samples presented a DNA concentration of 40.3–3213 ng/mL, low-risk samples presented a concentration of 332–2285 ng/mL, high-risk samples presented a concentration of 13.1–1852 ng/mL, and cancer samples presented a concentration of 67.6–2332 ng/mL. The results show significant differences between groups, with a lower DNA concentration in cancer than the other samples.

### 2.4. Western Blot Analyses of Proteins Isolated from Extracellular Vesicles from Bowel Lavage Fluid and Directly from Bowel Lavage Fluid

**Figure 5:**
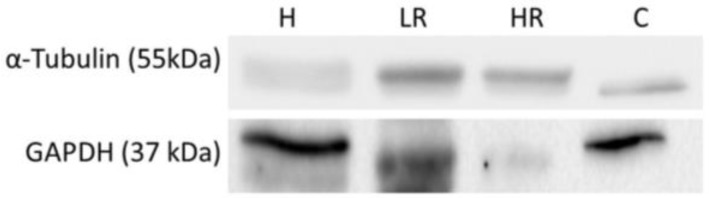
Representative bands and molecular weights of α-Tubulin and GAPDH expression levels directly determined in bowel lavage fluid by Western blot in healthy, low-risk, high-risk, and cancer samples. **H:** healthy samples; **LR:** low-risk samples; **HR:** high-risk samples; **C:** cancer samples.

Proteins can be isolated from EVs from BLF using TRI reagent LS (Table 2). Healthy samples presented a protein concentration of 1.75–74.4 µg/mL, low-risk samples presented a protein concentration of 5.10–31.3 µg/mL, high-risk samples presented a protein concentration of 5.33–58.8 µg/mL, and cancer samples presented a protein concentration of 1.60–41.6 µg/mL. The results show non-significant differences between groups. In addition, proteins can be directly determined from BLF (Table 2). Healthy samples presented a protein concentration of 84.0–1184 µg/mL, low-risk samples presented a protein concentration of 34.9–1302 µg/mL, high-risk samples presented a protein concentration of 54.1–1395 µg/mL, and cancer samples presented a protein concentration of 19.0–9994 µg/mL. The results show significant differences between groups, with a higher BLF protein concentration in cancer samples than the other samples. Finally, these proteins can be studied by Western blot (Figure 5).

## 3. Discussion

BLF has been demonstrated to have great potential as a research tool [16], since it has been used in various diseases, such as for enzyme research on colorectal polyps [21], for endoscopic screening for food allergies [22], CRC diagnosis [19], and molecular screening of inflammatory bowel diseases [23]. However, BLF is a rarely used sample with type with considerable potential for research [24] due to its proximity to the injured area and because this sample type does not cause extra discomfort to patients undergoing colonoscopy, since bowel preparation is the same as that required for a colonoscopy [16]. Nowadays, the use of EVs in liquid samples, such as saliva, amniotic fluid, breast milk, seminal liquid, nasal secretion, cerebrospinal fluid, lymph node secretion, urine, plasma, serum, placenta, bronchoalveolar fluid, synovial liquid, bile fluid, and ascites [9,25,26], is widespread, but there are no studies on EVs from BLF.

Despite this lack of studies, our results show that EVs can be isolated from BLF, as demonstrated by AFM and TEM results. Moreover, the NTA results demonstrate that these EVs presented a regular size, considering that exosomes have a diameter of 30–100 nm and microvesicles have a diameter of 100 nm^−1^ µm [27]. The size difference between TEM and NTA results can be attributed to the dehydrating conditions to which EVs are subjected during fixation for TEM analysis [8], in addition to the presence of a sufficient concentration of EVs in BLF, taking into account that in urine, the EV concentration determined by NTA is 1.00 × 10^10^ [28], whereas in CRC patient blood, the EV concentration is 1.29 × 10^9^ ± 9.92 × 10^8^ [29]. Finally, CD9 and CD63 expression, which are two tetraspanins considered EV biomarkers [8], has been found in EVs isolated from BLF. Altogether, these results suggest that EVs can be isolated from BLF.

EV cargo can be determined and studied, since RNA can be used to study gene expression levels [30], DNA mutations can be determined [31], and protein expression levels can be analyzed by Western blot and liquid chromatography–tandem mass spectrometry [29,32,33,34]. However, given the nature of our sample, it was necessary to confirm the content of these EVs from BLF as a feasible tool for CRC study. The RNA concentration results presented no differences between groups and demonstrated that the RNA concentration and quality are sufficient to determine gene expression levels, as can be seen in the *B2M* expression levels, which is an often used housekeeping gene due to its high and constant expression [35]. In addition, the protein concentration results in EVs did not present differences between groups, indicating an adequate concentration and quality for use for determination of protein expression levels, as can be seen in the CD9 and CD63 expression levels. However, the DNA concentration was lower in cancer samples. In contrast to our results, Bryzgunova and collaborators demonstrated that EVs of urine samples from patients with prostate cancer presented higher DNA concentrations than healthy samples [36], although these variances could be attributed to the different type of sample and cancer from which EVs were isolated. Despite this, DNA from EVs can be studied [31], and our results indicate a sufficient concentration of DNA for use, as shown in studies by Thakur and collaborators, who found that DNA concentration in serum EVs was 10.59 ± 13.19 ng/mL [37], or in studies by Choi et al., who determined that the amount of DNA in plasma EVs ranged from 0.1 to 2.48 ng [38]. Finally, protein expression levels can be directly determined in BLF samples, as shown in studies by Kayazawa and collaborators, in which lactoferrin, polymorphonuclear neutrophil elastase, myeloperoxidase, and lysozyme concentrations were determined in BLF samples by ELISA [39]. BLF protein concentrations in cancer samples; however Al-Muhtaseb and Bel’skaya demonstrated that in saliva from breast cancer patients, the total protein concentration was lower than that in control patients [40,41]; however these differences can could be attributed to the distinct sample and cancer types. Nevertheless, BLF protein presented an optimal concentration for use to determine protein expression levels, as shown by the α-Tubulin and GAPDH expression levels results, which are often-used proteins for Western blot analysis due to their high expression [42,43,44], demonstrating that this protein from BLF is suitable for Western blot determinations.

## 4. Materials and Methods

### 4.1. Patients and Sample Acquisition

A total of 170 patients (83 females with an average age 63.6 ± 11.4 and 87 males with an average age 63.8 ± 11.1) were included to carry out the study. Patients of both sexes received the a patient’s information sheet about the research project, and they signed an informed consent according to the “World Medical Association Declaration of Helsinki” for medical research involving humans. The study was approved by the *Comité d’Ètica de la Investigació de les Illes Balears* (IB 3833/19 PI).

Days prior to colonoscopy, patients received the information about colonoscopy and an information sheet about the research project. Then, 48 h before colonoscopy, patients were required to reduce fiber, fat, and gas intake, and 8 h before colonoscopy, patients drank 3 L of a polyethylene glycol solution to clean the bowel lumen. Colonoscopies were performed in the endoscopy unit at the *Hospital Comarcal d’Inca* with anesthetic sedation. BLF samples were recollected with saline solution (0.9% NaCl) applied directly to the injury area in the mucosa through an endoscopic flushing channel, then aspirated and retained in the polyp-trapping basket of the endoscope in order to avoids mixture with fluids from non-affected zones. The samples were stored at −80 °C and divided according to pathology: without pathology (healthy samples; *n* = 43), with polyps (divided into low- and high-risk of suffering from neoproliferative processes; *n* = 58 and *n* = 29, respectively), and cancer samples (*n* = 40). Low-risk samples correspond to 1 or 2 tubular adenomatous lesions with low-grade dysplasia and serrated lesions without dysplasia—all less than 10 mm; high-risk samples correspond to 3 or more tubular adenomatous lesions with low-grade dysplasia less than 10 mm, at least one adenomatous lesion with a villous component, high-grade dysplasia of more than 10 mm, and at least one serrated lesion with dysplasia or more than 10 mm [45].

### 4.2. Extracellular Vesicles Isolation by Ultracentrifugation

BLF samples were centrifuged for 15 min at 2000× *g* and 4 °C to eliminate debris and cellular components. Then, 4 mL of supernatant was separated, and 8 mL of sterile PBS 1× (500 mM NaCl, 167 mM NaH_2_PO_4_·2H_2_O, 333 mM Na_2_HPO_4_) pH 7.5 was added. Next, this mix was centrifuged at 2000× *g* for 10 min and 4 °C in order to remove the remaining debris. Finally, supernatants were centrifuged at 2000× *g* for 10 min at 4 °C to eliminate large particles. Subsequently, supernatants were centrifugated for 1 h at 4 °C and 100,000× *g* in order to precipitate EVs. The resulting pellets containing EVs were resuspended in 100 µL of sterile PBS 1× pH 7.5.

### 4.3. Atomic Force Microscopy (AFM)

First 50 µL of EV resuspension from healthy and cancer samples was added to a freshly cleaved muscovite mica surface (NanoAndMore GmbH, Wetzlar, Germany) for 10 min, washed with 2.5 mL of deionized water, and dried with nitrogen. Then, EVs were observed under an atomic force microscope (Veeco, Oyster Bay, NY, USA) in tapping mode with aluminum-coated silicon probe tips (HQ:NSC35/Al BS, Mikromasch, Lady’s Island, SC, USA). The height and amplitude of the samples were recorded at 512 pixels × 512 pixels at a scanning rate of 1 Hz and processed with NanoScope Image Software (v5.10, Veeco, Metrology, NY, USA).

### 4.4. Transmission Electron Microscopy (TEM)

EV resuspensions were observed by TEM following the protocol of Forteza-Genestra et al. [46]. Briefly, EV resuspensions were mixed 1:1 with 4% formaldehyde (F8775, Sigma-Aldrich, Darmstadt, Germany), and 10 µL of this mix was fixed on copper formvar–carbon-coated grids (Iesmat, Madrid, Spain) for 20 min. Then, grids were washed with PBS three times and incubated with 1% glutaraldehyde (G-7526, Sigma-Aldrich, Darmstadt, Germany) for 5 min. Finally, grids were washed with deionized water eight times. To visualize samples, grids were stained with 2% phosphotungstic acid for 1 min, then washed with deionized water for 1 min, and finally, air-dried. Images were taken with a Talos F200i transmission electron microscope (ThermoFisher, Madrid, Spain) at 20 kV and 50 kV.

### 4.5. Nanoparticle Tracking Analysis (NTA)

EV size distribution and particle concentration were analyzed using a Nanosight NS3000 (Malvern Instruments, Malvern, PA, USA). Samples were diluted 1:1000 in a final volume of 1 mL before analysis. Then, samples were passed through the chamber in vivo and recorded three times for 1 min each with a laser at a wavelength of 532 nm and an sCMOS camera. Finally, data were analyzed with NTA 3.2 Dev Build 3.2.16 software.

### 4.6. RNA Isolation, Reverse Transcription, and Real-Time PCR

Total RNA from EVs was isolated using TRI reagent LS^®^ (T3934, Sigma-Aldrich) following the manufacturer’s protocol. Briefly, TRI reagent was added to EV samples and left for 5 min at room temperature. Then, chloroform was added and incubated for 15 min at room temperature, followed by centrifugation at 12,000× *g* for 15 min at 4 °C. After centrifugation, three phases were differentiated. Isopropanol was added to the aqueous phase and incubated overnight at −20 °C for better RNA precipitation. After the incubation, samples were centrifuged at 12,000× *g* for 8 min at 4 °C, and the resulting pellets were washed with frozen 75% ethanol and centrifuged at 7500× *g* for 5 min at 4 °C. The supernatants were discarded, and the pellets were dried under vacuum. Finally, RNA was resuspended in 20 µL of RNase-free water and quantified using a BIO-TEK PowerWave XS spectrophotometer at wavelength of 260 nm. The RNA quality was checked by a 260/280 ratio.

The obtained RNA was mixed to create two or three RNA pools for each sample type to better represent each sample type, avoiding the particular patient characteristics. Then, 300 ng of the total RNA was reverse-transcribed to cDNA. First, a denaturalization at 90 °C for 1 min was applied to RNA samples. Next, the reverse transcription reagents were added to the sample (50 µM random hexamers (10609275, Fisher Scientific, Madrid, Spain), 2.5 mM dNTPs mix (100 mM dGTP solution 10218-014, 100 mM dTTP solution 10219-012, 100 mM dATP solution 10216-018, and 100 mM dCTP solution 10217-016; Fisher Scientific), 20 U/µL RNase Out (10615995, Fisher Scientific), 0.1 M DTT, buffer 5×, and 200 U/µL M-MLV (10338842, Fisher Scientific)) and incubated at 25 °C for 10 min, then at 37 °C for 50 min, 70 °C for 15 min, and finally, at 4 °C. cDNA aliquots were frozen at −20 °C after 1/10 dilution in RNase-free water.

A LightCycler 480 System II rapid thermal cycler (Roche Diagnostics, Basel, Switzerland) with SYBR Green technology was used to carry out the real-time PCR, following the manufacturer’s protocol. The expression of beta-2-microglobulin (*B2M*) was analyzed (forward primer: 5′-TTT CAT CCA TCC gAC ATT GA-3′; reverse primer: 5′-Cgg CAg gCA TAC TCA TCT TT-3′; accession number: NM_004048). The first step in the amplification program was preincubation for cDNA denaturation at 95 °C for 5 min, followed by 50 cycles of denaturation at 95 °C for 10 s, annealing at 54 °C for 10 s, and elongation at 72 °C for 12 s. Finally, melting was applied at 95 °C for 5 s, followed by 65 °C for 1 min, and 97 °C continuously until cooling at 40 °C.

### 4.7. DNA Isolation and Quantification

DNA from EVs was isolated using TRI reagent LS^®^ (T3934, Sigma-Aldrich) following the manufacturer’s protocol. Briefly, after the formation of three phases, 100% ethanol was added to the interphase and organic phase, mixed, and incubated for 3 min at room temperature. Next, samples were centrifuged at 2000× *g* for 5 min at 4 °C, and the supernatants were saved in a new tube for protein isolation. The pellets were mixed with 0.1 M trisodium citrate in 10% ethanol and incubated for 30 min at room temperature, followed by centrifugation at 2000× *g* for 5 min at 4 °C; this step was repeated twice. Then, pellets were washed with 75% ethanol and incubated for 20 min at room temperature, followed by centrifugation at 2000× *g* for 5 min at 4 °C. The resulting pellets were dried at room temperature for 15 min and resuspended in 100 µL of 8 mM NaOH. Finally, centrifugation was performed at 12,000× *g* for 10 at 4 °C, and supernatants were saved. Finally, DNA was quantified using a BIO-TEK PowerWave XS spectrophotometer at a wavelength of 260 nm. The DNA quality was checked by a 260/280 ratio.

### 4.8. Protein Isolation and Quantification

Protein from EVs was isolated using TRI reagent LS^®^ (T3934, Sigma-Aldrich) following the manufacturer’s protocol. Briefly, the supernatants saved during DNA isolation were incubated with isopropanol at room temperature for 10 min and centrifuged at 12,000× *g* for 10 min at 4 °C. The supernatants were discarded, and precipitates were washed three times with 0.3 M guanidine hydrochloride in 95% ethanol, incubated at room temperature for 20 min, and centrifuged at 7500× *g* for 5 min at 4 °C. After three washes, 100% ethanol was added to the precipitates and incubated at room temperature for 20 min, followed by centrifugation at 7500× *g* for 5 min at 4 °C. Next, protein pellets were air-dried for 15 min, dissolved in 100 µL of 1% SDS, and incubated overnight at −20 °C. Then, samples were centrifuged at 10,000× *g* for 10 min at 4 °C, and the supernatant was transferred to a new tube. Finally, protein was quantified by a Pierce^®^ BCA protein assay kit (10741395, Fisher Scientific) following the manufacturer’s protocol.

Protein from BLF samples was directly quantified by the Bradford method [47].

### 4.9. Western Blot

For all SDS-PAGE assays carried out for EV–protein, 5 µg of total protein was loaded; for all SDS-PAGE assays carried out for BLF–protein, 10 µg of total protein was loaded. First, loading buffer (0.25 M Tris-HCl (pH 6.8), 10% SDS, 40% glycerol, and 0.1% bromophenol blue; for BLF protein, 10% fresh β-mercaptoethanol was added) was added to each sample. Then, samples were boiled for 5 min. Protein was separated by electrophoresis on 12% acrylamide/bisacrylamide (30/1) gels. Next, proteins were electrotransferred onto a 0.2 µm nitrocellulose membrane (Bio-Rad Laboratories, Hercules, CA, USA) using a trans-blot turbo transfer system (Bio-Rad Laboratories, CA, USA). After electrotransfer, membranes were incubated with Ponceau staining for equal sample loading validation. Then, membranes were blocked with 5% non-fat powdered milk in Tris-buffered saline 0.05%–Tween 20 pH 7.6 (TBS-Tween) for 1 h at room temperature under agitation. After blocking, membranes were washed with TBS–Tween and incubated with primary antibody (5% BSA and 0.05% sodium azide in TBS–Tween) overnight at 4 °C under agitation. The following primary antibodies used and dilutions were used: 1:500 α-tubulin (sc-5286, Santa Cruz Biotechnology, Dallas, TX, USA), 1:1000 GAPDH (sc-365062, Santa Cruz Biotechnology, Dallas, TX, USA), 1:1000 CD9 (Ts9, 10626D, ThermoFisher, Spain), and 1:5000 CD63 (Ts63, ab59479, Abcam, Boston, MA, USA). After primary incubation, membranes were washed with TBS–Tween and incubated with horseradish-peroxidase-conjugated secondary antibody and 1:10,000 anti-mouse antibody (A9044, Sigma-Aldrich, Darmstadt, Germany) in 2% non-fat powdered milk in TBS–Tween for 1 h at room temperature under agitation. Then, membranes were washed with TBS–Tween, and TBS and immunoreactivity was detected by an Inmun-Star© Western chemiluminescence kit and Western blotting detection system (Bio-Rad Laboratories, Hercules, CA, USA). Chemidoc Imaging System (Bio-Rad Laboratories, Hercules, CA, USA) was used to acquire the chemiluminescent signal.

### 4.10. Statistical Analysis

Statistical Program for the Social Sciences software for Windows (SPSS, version 25.0; SPSS Inc., Chicago, IL, USA) was used to perform all statistical analyses. First, a boxplot was used to discard the outliers. Then, a normality study was performed using the Shapiro–Wilk test; for parametric results (EV size, EV concentration, and *B2M* Ct values) a one-way ANOVA was used to analyze differences between groups; for non-parametric results (RNA, DNA, EV and BLF protein concentrations, and *B2M* Tm values), the Kruskal–Wallis test was used to analyze differences between groups. All determinations were made with minimal statistical significance at *p* < 0.05, and all results are presented as mean ± SD.

## 5. Conclusions

In conclusion, bowel lavage fluid is a sample that must be taken into account in the study of colorectal cancer due to its proximity to the tumor. In addition, the extracellular vesicles isolated from this sample type can be useful as a source of colorectal cancer biomarkers, considering that EV content can be determined and studied by different molecular biology techniques. The possibility of studying the content of extracellular vesicles isolated from bowel lavage fluid could improve knowledge of colorectal cancer, in addition to identifying new biomarkers (for diagnosis, prognosis, and monitoring of the disease), which could be extrapolated to non-invasive samples, such as stool samples. Nevertheless, further investigation of bowel lavage fluid and, specifically the extracellular vesicles from such samples, is necessary to better understand the mechanism whereby EVs are released from cancer cells and the role that their content plays in cancer initiation, progression, and metastasis.

## Figures and Tables

**Table 1:** Extracellular vesicle concentration (particles/mL) in healthy, low-risk, high-risk, and cancer samples determined by nanoparticle tracking analysis. Values are represented as mean ± SD. **NS**: non-significant differences (*p* 0.690), (ANOVA; *p* < 0.05). *n* = 48; for each sample type *n* = 12 divided into 3 pools of 4 samples each.

	Healthy	Low-Risk	High-Risk	Cancer	ANOVA
Particles/mL	1.80 × 10^10^ ± 7.80 × 10^9^	1.36 × 10^10^ ± 5.57 × 10^8^	1.51 × 10^10^ ± 3.59 × 10^9^	1.59 × 10^10^ ± 2.12 × 10^9^	NS (*p* 0.690)

**Table 2:** Concentration of extracellular vesicle RNA (ng/mL BLF), extracellular vesicle DNA (ng/mL BLF), extracellular vesicle protein (µg/mL BLF), and direct protein from bowel lavage fluid (BLF protein; µg/mL BLF) in healthy, low-risk, high-risk, and cancer samples. *n* ≥ 17; values are represented as mean ± SD. **S**: significant differences (*p* 0.000 for EV DNA; *p* 0.003 for BLF protein); **NS**: non-significant differences (*p* 0.431 for EV RNA; *p* 0.149 for EV protein), (Kruskal–Wallis; *p* < 0.05).

	Healthy	Low-Risk	High-Risk	Cancer	Kuskal–Wallis
ng/mL BLF of EV RNA	707 ± 844	225 ± 187	434 ± 585	1791 ± 2744	NS (*p* 0.431)
ng/mL BLF of EV DNA	1055 ± 786	800 ± 482	756 ± 420	477 ± 547	S (*p* 0.000)
µg/mL BLF of EV Protein	21.7 ± 17.1	14.0 ± 9.12	18.0 ± 12.1	15.5 ± 12.2	NS (*p* 0.149)
µg/mL BLF of BLF Protein	392 ± 253	387 ± 249	407 ± 322	1487 ± 2179	S (*p* 0.003)

**Table 3:** B2M Ct and Tm values in healthy, low-risk, high-risk, and cancer samples determined by real-time PCR. Values are represented as mean ± SD. **NS**: non-significant differences (*p* 0.108 for Ct values; *p* 0.522 for Tm values) (Ct values ANOVA, *p* < 0.05; Tm values Kruskal–Wallis, *p* < 0.05).

	Healthy	Low-Risk	High-Risk	Cancer	Statistics
Ct values	32.9 ± 1.87	28.4 ± 1.09	32.0 ± 1.74	31.2 ± 0.98	ANOVA: NS (*p* 0.108)
Tm values	81.5 ± 0.09	81.4 ± 0.04	81.5 ± 0.01	81.5 ± 0.02	Kruskal-Wallis: NS (*p* 0.522)

## Data Availability

The data presented in this study are available in this article.
